# Predicting Facial Attractiveness from Colour Cues: A New Analytic Framework

**DOI:** 10.3390/s24020391

**Published:** 2024-01-09

**Authors:** Yan Lu, Kaida Xiao, Michael Pointer, Yandan Lin

**Affiliations:** 1 School of Design, University of Leeds, Leeds LS2 9JT, UK; yan.lu@manchester.ac.uk (Y.L.); mrpointer@btinternet.com (M.P.); 2 Department of Electrical & Electronic Engineering, University of Manchester, Manchester M13 9PL, UK; 3 School of Information Science and Technology, Fudan University, Shanghai 200433, China; ydlin@fudan.edu.cn

**Keywords:** facial attractiveness, skin colour, multivariate regression, machine learning, predictive accuracy

## Abstract

Various facial colour cues were identified as valid predictors of facial attractiveness, yet the conventional univariate approach has simplified the complex nature of attractiveness judgement for real human faces. Predicting attractiveness from colour cues is difficult due to the high number of candidate variables and their inherent correlations. Using datasets from Chinese subjects, this study proposed a novel analytic framework for modelling attractiveness from various colour characteristics. One hundred images of real human faces were used in experiments and an extensive set of 65 colour features were extracted. Two separate attractiveness evaluation sets of data were collected through psychophysical experiments in the UK and China as training and testing datasets, respectively. Eight multivariate regression strategies were compared for their predictive accuracy and simplicity. The proposed methodology achieved a comprehensive assessment of diverse facial colour features and their role in attractiveness judgements of real faces; improved the predictive accuracy (the best-fit model achieved an out-of-sample accuracy of 0.66 on a 7-point scale) and significantly mitigated the issue of model overfitting; and effectively simplified the model and identified the most important colour features. It can serve as a useful and repeatable analytic tool for future research on facial impression modelling using high-dimensional datasets.

## 1. Introduction

Facial attractiveness plays a pivotal role in shaping human perceptions, influencing societal interactions, and driving decision making across various domains [1,2]. Despite the significance of facial structures [3,4,5], more recent research increasingly highlights the influential role of facial colour cues, encompassing skin tone, colour variations, and contrasts, on attractiveness evaluations, often associated with perceptions of health, age, and vitality. For instance, overall facial redness, yellowness, and lightness are most widely examined as cultural-specific determinants of attractiveness perception [6,7,8,9,10]. The colouration of specific regions, such as cheeks, periorbital area, and lips, has also been identified as a valid predictor of facial attractiveness [11,12]. Furthermore, more uniform skin colour or accentuated colour contrasts between facial features like eyes, brows, and mouth relative to the surrounding skin are both correlated positively with perceived attractiveness [13,14,15,16,17]. A summary of these potential colour cues of facial attractiveness is provided in the materials and methods section.

The axiom in most previous research described above is to change a single colour variable in a controlled experiment for attractiveness evaluation. Such isolation, however, neglects the holistic and complex nature of attractiveness judgement upon real human faces and makes it impossible to assess the relative importance of different colour variables in real scenarios. Meanwhile, the manipulation could easily make the colour out of the ecologically valid range. In contrast, the recent growing body of work has shifted towards the use of realistic skin models in experiments for face perception or preference evaluation without any image manipulation [13,18,19,20,21,22,23]. The problems of the univariate experimental approach and the necessity of using realistic models for mapping physical characteristics to facial impressions or preference judgements have been emphasised [24,25].

Currently, even research employing realistic skin models could underestimate the role of colour in preference judgements, as very limited colour characteristics (usually, the overall skin tone) have been considered together with many other physical or biophysical properties of the face [13,18,20,21]. Little is known about the impact of diverse colour cues taken together on facial attractiveness judgment. Our recent work has also confirmed the limited role of the overall skin tone in preference judgements on real human faces, but efforts have been made to show there are stronger links between colour and facial preference when more variables are included [22,23]. In the current study, a larger set of facial images of real human faces was used with colour rigorously controlled. Colour analyses were performed on each of the images to extensively include potential colour cues of attractiveness.

Due to the high number of candidate colour variables and possible inherent correlations between them, predicting attractiveness from various colour cues could be difficult and may depend on the modelling methods. An important challenge is to select valid predictors from a large number of variables and avoid the problem of multicollinearity and overfitting. Despite its difficulty, there is a strong need to predict facial preference in many applications such as cosmetics, medical and aesthetic surgeries, lighting, and the imaging industry [26,27,28]. From a practical perspective, it is often desirable to achieve good predictive accuracy with fewer explanatory variables. Thus, different multivariate approaches were tested in this study regarding their fitness in facial attractiveness modelling. To avoid the issue of overfitting, an independent testing dataset of attractiveness evaluation was collected to validate the out-of-sample predictive accuracy of different modelling methods.

Multivariate regression techniques are useful for modelling the relationship between a large set of explanatory variables and the response variable. Multiple linear regression is one of the most popular multivariate statistical techniques utilised for forecasting in diverse fields such as the environmental [29,30,31], economics [32], psychology [33], and facial impression [13,18,19]. Running the full model with too many variables, especially irrelevant ones, will lead to a needlessly complex model. To simplify the model, subset selection, such as stepwise regression, is used to incrementally incorporate or exclude predictors based on certain criteria (e.g., AIC, correlation significance, etc.), ensuring only vital variables remain [34]. Two other strategies have been proposed to address the issue of multicollinearity. The first reduces the dimensionality of the data matrix before modelling, using methods like principal component regression (PCR) and partial least squares regression (PLSR). The method overcomes the problem of multicollinearity by projecting the original predictors into an uncorrelated subspace of principal components or latent components and then fitting the linear regression model [35,36,37]. The second strategy utilises a technique from ML, regularisation or penalisation, to constrain the coefficients of a model and reduce the variance of the parameter estimators. The most well-known regularisation regression methods are ridge, lasso, and elastic net. Ridge regression (RR) still maintains the structure of the linear regression model but shrinks the coefficients to approach 0 [38,39]. Least absolute shrinkage and selection operator regression (LASSO) penalises high coefficients of the informative variable to be 0 [40]. Elastic net regression (EN) combines shrinkage from RR and LASSO and balances the two algorithms by weighting the two effects [41,42]. The implementation of this approach in facial impression research can be found in previous work [43,44]. The current study assessed all the strategies mentioned above in terms of their effectiveness in attractiveness modelling from colour traits.

Using datasets from Chinese subjects, this study aimed to establish a new analytic framework for attractiveness modelling based on colour cues. The framework was designed to address the limitations in prior research: (1) the univariate experimental approach was avoided; instead, a large set of realistic skin models was used to extract facial colour features with ecologically valid variations; (2) an extensive range of colour characteristics was included, allowing a more comprehensive evaluation; and (3) the issue of inherent correlations and potential model overfitting was addressed by comparing various statistical and machine learning regression techniques and using a novel testing dataset, which played a crucial role in validating the predictive accuracy of different models. The proposed methodology effectively improved predictive accuracy and could serve as a useful and repeatable analytic tool for future studies on robust facial impression modelling from a high-dimensional dataset of facial features.

## 2. Materials and Methods

### 2.1. Facial Images

One hundred images of real human faces were used as experimental materials. Those images were all Chinese facial images selected from the Leeds Liverpool Skin Colour Database (LLSD), with neutral facial expressions and mid-grey backgrounds [45]. The image dataset was captured by the authors using a Nikon D7000 (Nikon Corporation, Tokyo, Japan) and a Canon EOS 6D Mark II (Canon Inc., Tokyo, Japan) DLSR camera. The images were all captured under standard lab conditions and then processed based on rigorous colour management procedures with the purpose of accurately measuring and reproducing the colour appearance of human faces. The details of the photograph and colour management have been described in previous work [22]. The colour-calibrated images were used in experiments for attractiveness evaluation as representations of the naturally occurring facial colour variations in Chinese populations.

### 2.2. Facial Attractiveness Evaluation

Two separate sets of facial attractiveness evaluation data were collected through psychophysical experiments in Leeds, UK, and Shanghai, China, respectively. The experimental procedure and data collection method at the two sites were the same: the experiments were conducted in a dark room using a calibrated display; the observers viewed each of the facial images presented in a random order and then made a categorical judgement of perceived facial attractiveness based on the skin colour using a 7-point Likert-type scale, where 1 represented ‘least attractiveness’ and 7 represented ‘best attractiveness’. Experiment 1 was conducted in the UK and had 40 Chinese images assessed by a panel of Chinese observers. In order to validate the out-of-sample predictive accuracy of different models, an independent testing dataset was collected in China (Experiment 2) using a new set of 60 Chinese images and a new panel of Chinese observers. The same 40 images used in Experiment 1 were also assessed in Experiment 2 to test observer consistency between the two sites.

Experiment 1 used twenty-two Chinese observers (7 males; mean age ± SD = 26.05 ± 3.96) with normal colour vision. They were all native Chinese and, at the time of study, either students or visiting scholars at Leeds University with 1–3 years’ living experience in the UK. Experiment 2 had fifty-one native Chinese observers (21 males, mean age ± SD = 24.45 ± 4.10) with normal colour vision who participated. They were either students or researchers at Fudan University, China.

### 2.3. Proposed Analytic Framework

**Figure 1:**
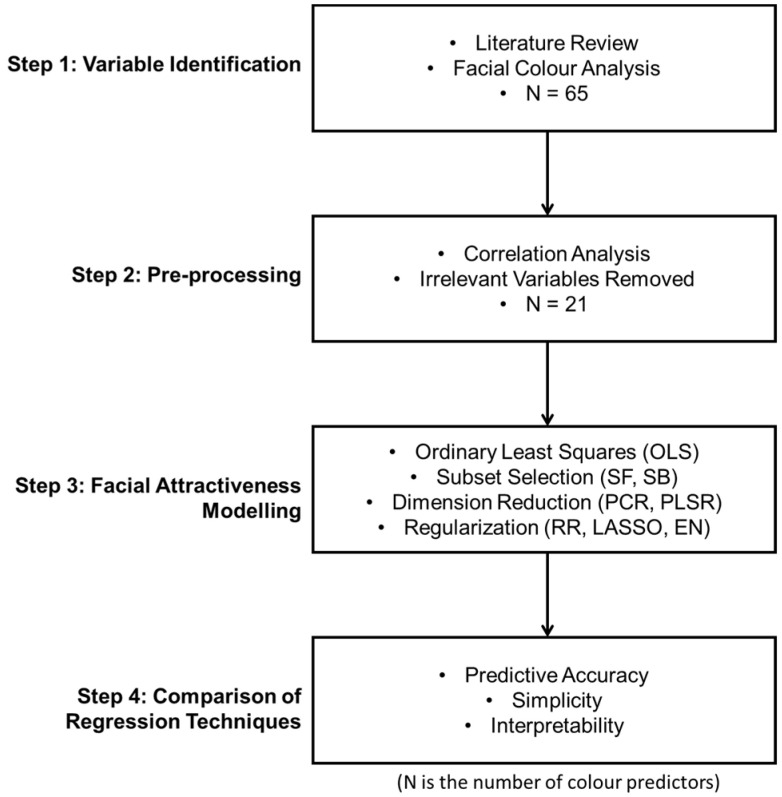
The proposed analytic framework.

This research presented a new data analytic framework for facial attractiveness modelling from colour cues, as shown in Figure 1. The main motivation for building this new framework was to reveal the importance of various facial colour cues under more realistic conditions and to address model overfitting issues due to the high number of candidate variables and their correlations.

A four-step analysis procedure was followed. The first step was to identify all the potential colour predictors of attractiveness. Table 1 summarises the total of sixty-five explanatory colour variables included in this study, the corresponding method of calculation, and the references. Analyses of those facial colour characteristics were performed on each of the facial images. The Pearson Correlation Coefficient (two-tailed) was used to assess the relationships between the attractiveness rating and various colour variables. Based on the correlation matrix, the second step was to select effective predictors and remove the irrelevant ones (having non-significant correlations with attractiveness ratings, *p* > 0.05) from a large number of variables. Facial attractiveness ratings were then modelled from the relevant colour characteristics in step 3 using the eight multivariate statistical and ML regression techniques. In the last step, a model comparison was conducted across different regression techniques using three criteria, predictive accuracy, simplicity, and interpretability. The regression techniques with best model performance were recommended. The effectiveness of this proposed framework was also validated based on the model comparison between the conventional univariate models and the recommended multivariate approaches.

#### 2.3.1. Facial Colour Analysis

**Figure 2:**
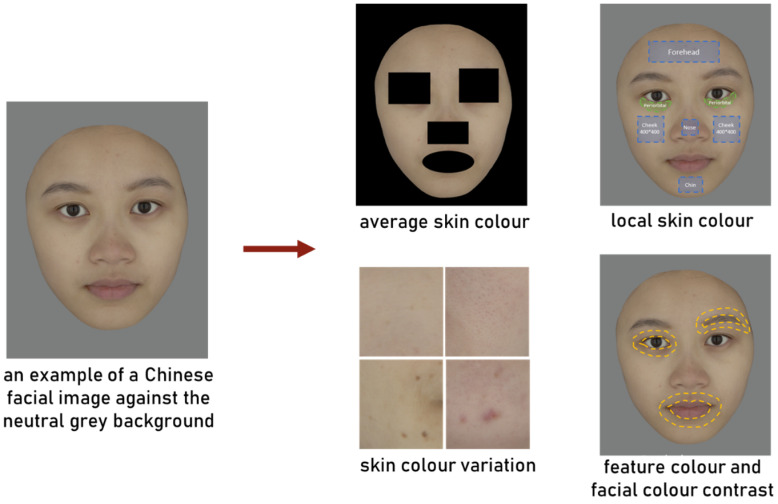
An example of a Chinese facial image and the areas selected for calculating different facial colour characteristics.

Analyses of the 65 facial colour characteristics listed in Table 1 were performed on each of the facial images, as in the example shown in Figure 2. Based on the digital camera colour characterisation, the colour specifications of each pixel were obtained and various facial colour characteristics were accurately calculated in a device-independent standard CIELAB colour space [46]. Five categories of facial colour cues, as revealed in previous studies, were measured in terms of the three CIELAB coordinates (L*, a*, b), together with the chroma, C*, and hue angle, h_ab_, as they may also be important colour parameters in relation to perception. The method of image analysis and calculation, explanatory variables, and references of each colour category are detailed in Table 1. Figure 2 provides a clear outline of the specific facial regions where those colour analyses were performed.

**Table 1:** Summary of facial colour characteristics included in this study.

Category	Method	Explanatory Variables	References
Average skin colour	The overall mean colour value of all pixels within the facial skin area.	L*, a*, b*, C*, h_ab_	[6,7,10,19,47,48,49]
Local skin colour	The mean colour values of the pixels within the five local facial skin areas: forehead, cheek, nose, chin, and periorbital.	Forehead_L*, a*, b*, C*, h_ab_	[11]
		Cheek_L*, a*, b*, C*, h_ab_	
		Nose_L*, a*, b*, C*, h_ab_	
		Chin_L*, a*, b*, C*, h_ab_	
		Periorbital_L*, a*, b*, C*, h_ab_	
Feature colour	The mean colour values of the pixels within the three facial feature areas: lip, brows, and eyes.	Lip_L*, a*, b*, C*, h_ab_	[12]
		Brows_L*, a*, b*, C*, h_ab_	
		Eyes_L*, a*, b*, C*, h_ab_	
Skin colour variation	The mean colour difference from the mean (MCDM) of the forehead, cheek, nose, chin, and overall facial skin area [50,51].	MCDM_Forehead	[13,14,16,52]
		MCDM_Cheek	
		MCDM_Nose	
		MCDM_Chin	
		MCDM	
Facial colour contrast	The adapted version of Michelson contrasts between three facial features (eyes, eyebrows, and mouth) and their surrounding skin [53].	Eyes_C_L*, a*, b*, C*, h_ab_	[17,54,55,56,57]
		Brows_C_L*, a*, b*, C*, h_ab_	
		Mouth_C_L*, a*, b*, C*, h_ab_	

#### 2.3.2. Multivariate Regression Techniques

The data collected in Experiment 1 were used as a training dataset for all model estimations. The explanatory variables were normalised to have zero mean and unit standard deviation before modelling. Based on high observer consistency, ratings were averaged across all observers to create a score for each face before modelling from the face level colour traits.

Classic ordinary least squares regression (OLS) and seven strategies for robust regression of high-dimensional datasets were used to predict attractiveness from facial colour cues. The OLS was included for comparison, where all the relevant colour predictors were involved in one model without any process of variable selection. The other seven strategies proposed were based on the three most commonly used multivariate techniques, subset selection, dimension reduction, and regularisation. For the regression techniques that have tuning parameters, a ten-fold cross-validation was performed to determine the optimal parameters with the maximised model fit and to optimise the algorithms. All the analyses were carried out in R (RDC, 2010).

- Subset selection regression

Forward stepwise (SF) and backward stepwise (SB) methods were tested and the subset selection was achieved by an iterative procedure based on Akaike Information Criterion (AIC) [34,58]. SF starts with intercept and adds colour predictors based on AIC in a stepwise manner, while SB starts with a full model and removes colour predictors in a stepwise manner until AIC is no better. The stepwise regression was implemented using the *olsrr* package.

- Dimension reduction regression

Two-dimension reduction regressions, principal component regression (PCR), and partial least squares regression (PLSR) were considered. In PCR, a ten-fold cross-validation was utilised to determine the number of principal components by minimising the root mean squared error (RMSE) of the prediction on the one-fold new data. In PLSR, the ten-fold cross-validation was also adopted to select the optimal number of latent components. PCR and PLSR were implemented using the *pls* package.

- Regularisation regression

Three regularisation regression methods were adopted: ridge regression (RR), lasso regression (LASSO), and elastic net (EN). A ten-fold cross-validation process was used to define the shrinkage parameter, lambda (while alpha = 0), of RR and control how aggressively the coefficients were shrunk toward zero. In LASSO, the same ten-fold cross-validation process was performed to determine the lambda (while alpha = 1). In EN, both parameters, alpha and lambda, can be tuned to optimise the model fit where alpha controls the degree to which the model shrinks coefficients and lambda determines how aggressively coefficients are set to zero. The ten-fold cross-validation was again implemented to generate the best combination of alpha and lambda with the maximised fit (minimised RMSE). The regularisation regressions were implemented using the *glmnet* and *caret* packages.

#### 2.3.3. Model Comparisons

Different multivariate regression techniques were compared in terms of their predictive accuracy, simplicity, and interpretability. Both the in-sample and the out-of-sample predictive accuracy were measured using the training dataset collected in Experiment 1 and the novel testing dataset collected in Experiment 2 (a new set of images and a new panel of observers), respectively. Root mean square error (RMSE) was adopted as a measure of predictive accuracy, which calculates the difference between the observed values (attractiveness scores rated by observers) and modelled values (attractiveness scores predicted by models). The coefficients of determination R^2^ show the goodness of fit, calculated as the square of the Person correlation coefficient between the observed values and modelled values. By contrast with R^2^, RMSE is not inflated by the number of predictors and has the same unit as the original scale used in the experiments. Meanwhile, the number and selection of colour predictors in each model were considered. For the regression techniques that perform direct variable selection, such as SF, SB, LASSO, and EN, the model was also evaluated by the number and selection of colour predictors that remained in the model. For the rest of the methods including OLS, PCR, PLSR, and RR, all the variables remained in the model and the rank of the variables was compared according to the standardised regression coefficients.

To evaluate the effectiveness of the proposed framework, the recommended multivariate approach was also compared with the conventional univariate models. An additional simulation analysis was conducted to assess the impact of the number of variables (N) on the model performance. For each N, a corresponding set of colour variables was randomly selected from the larger set of relevant predictors. These variables were then utilised to construct the model, and the resulting root mean square error (RMSE) was calculated to evaluate model performance. The RMSE was determined for both in-sample (training) and out-of-sample (testing) datasets. To ensure robustness and account for variability, this random selection and model building process was iterated 30 times for each value of N. The resulting RMSEs were then aggregated to calculate the mean and standard deviation, providing a measure of the model’s accuracy and consistency across different iterations.

## 3. Results

### 3.1. Observer Consistency

Cronbach’s alpha coefficient was calculated as a measure of inter-observer variability for attractiveness ratings across observers [59,60]. The internal consistency was very high for attractiveness ratings in both Experiment 1 (Cronbach’s alpha = 0.96, 95% CI [0.94, 0.98]), and Experiment 2 (Cronbach’s alpha = 0.98, 95% CI [0.98, 0.99]).

The rating results of the same forty Chinese images used in both experiments were compared to assess the consistency between the two groups of Chinese observers, and the two sets of rating scores were found to be significantly highly correlated (Person’s correlation coefficient: r(38) = 0.94 [0.89, 0.97], *p* < 0.001). The high consistency of the preference judgement results between the two experiments showed that the short experience of living abroad would not affect the aesthetic preference of Chinese observers.

### 3.2. Correlation Matrix

**Figure 3:**
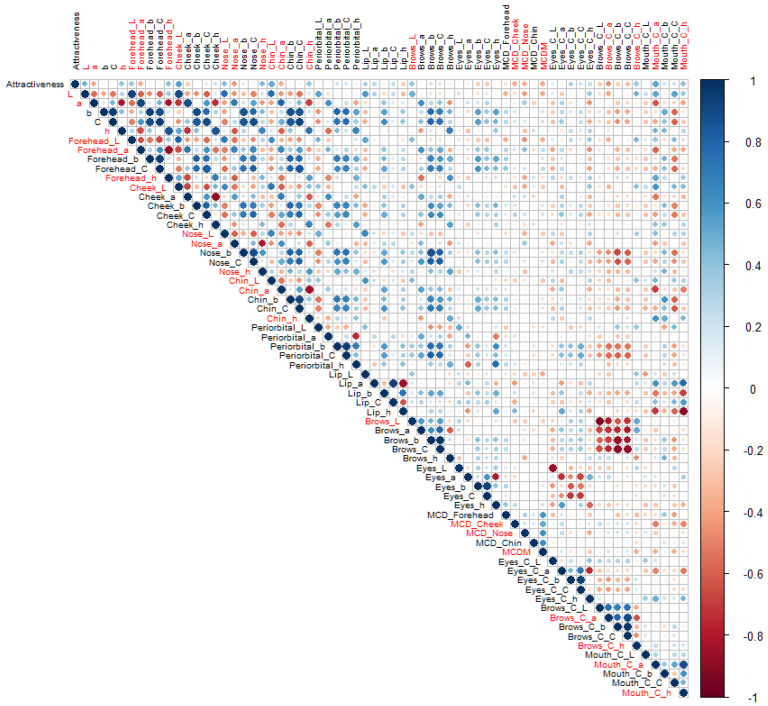
The correlation matrix between facial attractiveness ratings and facial colour characteristics. The twenty-one colour variables that have significant correlations (*p* < 0.05) with attractiveness ratings are marked in red.

Figure 3 shows the correlation matrix between the attractiveness ratings and all the facial colour characteristics, and the colour variables that have significant correlations (*p* < 0.05) with attractiveness ratings are marked in red. Among the five different colour coordinates, all the significant colour cues were basically related to skin lightness (L*), redness (a*), and hue angle (h_ab_) rather than yellowness (b*) or chroma (C*). The twenty-one significant colour characteristics were then used as valid explanatory variables in the next step of the mathematical modelling. Note that some of these facial colour characteristics were correlated with each other, e.g., the local skin lightness (L*) was often highly correlated with the average skin lightness (L*) of the same faces. Further variable selection was carried out during the modelling process and the collinearity was considered when building the models.

### 3.3. Comparisons of Multivariate Regression Techniques

#### 3.3.1. Predictive Accuracy and Model Fit

The results of the in-sample and out-of-sample model performance of the eight regression methods are shown in Table 2. Differences in RMSE values between different models were relatively smaller for the training dataset (from 0.42 to 0.62) but larger for the testing dataset (from 0.66 to 1.35). The range of R^2^ for the training dataset across different models was from 42.6% to 73.9% and is always lower on the test, as expected. OLS showed the lowest RMSE and highest R^2^ value for the training dataset; it performed the worst on the testing dataset. In this study, PCR selected two principal components for optimal model fit, and the two components explained 58.6% of the variance in the original predictors and 42.6% of the variance in attractiveness; PLSR selected only one component, which explained 41.5% of the variance in the original predictors and 44.8% of the variance in attractiveness. Regularisation techniques achieved better out-of-sample model performance than all the other models, where EN showed the lowest RMSE value and RR had the highest R^2^ value for the testing dataset.

**Figure 4:**
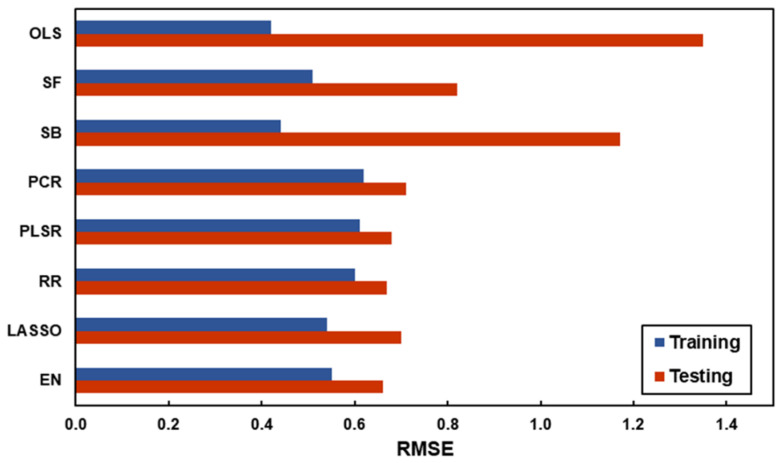
The RMSE values of the eight regression models in predicting facial attractiveness for the training data (blue bars) and the testing dataset (red bars).

The predictive accuracy of the eight regression models in predicting facial attractiveness is also demonstrated in the bar plots in Figure 4. The OLS and subset selection regressions showed lower in-sample RMSE values but higher out-of-sample RMSE values. The models using dimension reduction and regularisation techniques were just the opposite, resulting in closer RMSE values between the training dataset and the testing dataset. The scatter plots in the Supplementary Materials give the comparisons between the actual values of facial attractiveness ratings recorded during the experiments and the predicted values of facial attractiveness calculated from the regression models of each of the facial images (each red data point in the left column represents one of the forty facial images that were judged in Experiment 1, and each blue data point in the right column represented one of the sixty facial images that were judged in Experiment 2). The different degrees of dispersion of the testing dataset also indicate the different out-of-sample model performances as mentioned above.

#### 3.3.2. Ranking and Selection of Predictors

The numbers of colour predictors that were selected by the SF, SB, LASSO, and EN models were 7, 11, 11, and 14, respectively. For the OLS, PCR, PLSR, and RR models, all 21 variables remained in the model. Based on the standardised regression coefficients in all regression models, the colour predictors were ranked in each model. Considering that the regularisation techniques gave the better predictive accuracy as described in the previous section, the top 11 colour predictors selected by both LASSO and EN are listed in Table 3, ordered by the relative importance of each colour predictor (according to absolute standardised regression coefficients) in the EN model (last column). The rankings of these 11 colour predictors in eight regression models are given in the table. Some variables that were not selected by SF or SB are marked as 0. Between RR, LASSO, and EN, almost all the top 11 variables selected were held in common.

**Figure 5:**
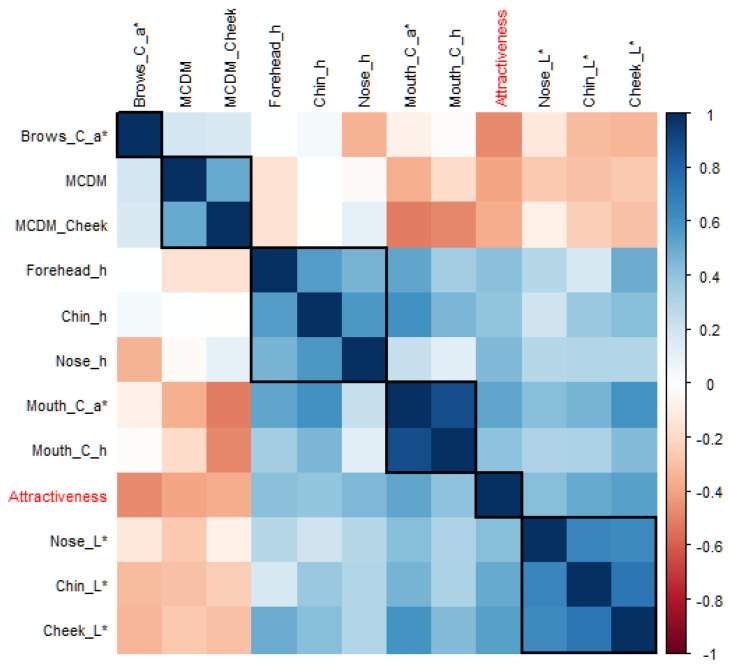
The correlation matrix between the eleven colour predictors selected by LASSO and EN and facial attractiveness ratings.

The correlation matrix between the top eleven colour predictors selected by LASSO and EN and facial attractiveness ratings are further visualised in the heatmap in Figure 5. To identify the hidden structure and pattern in the matrix, the 11 colour predictors were reordered based on the hierarchical clustering as shown in the five black boxes, which were brows colour contrast (a*), skin colour variation (overall or cheek), local skin hue angle (forehead, chin, or nose), the mouth colour contrast (a*), and local skin lightness L* (nose, chin, or cheek), from top left to bottom right.

### 3.4. The Effect of the Number of Variables on Predictive Accuracy

**Figure 6:**
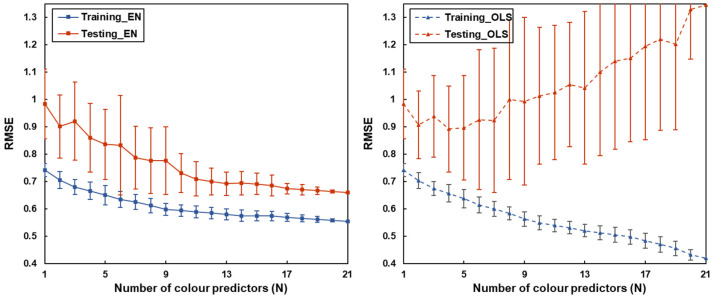
The effect of the number of colour predictors (N) on RMSE values of the EN (**left**, solid line) and OLS (**right**, dash line) models in predicting facial attractiveness for the training data (blue line) and the testing dataset (red line). The error bars denote the standard deviation of RMSE across the random 30 iterations.

To further assess the impact of variable number on predictive accuracy, a comparative analysis of RMSE trends with an increasing number of colour predictors was performed (Figure 6). Based on the results above, the regression technique with the highest out-of-sample accuracy and relatively smaller number of selected predictors, the EN model, was used in comparison with the conventional OLS model. For each selected number of predictors (N), a corresponding set of N colour predictors was randomly selected from the set of relevant colour predictors to build the model. Figure 6 shows the mean and standard deviation of RMSEs across 30 random iterations.

Noticeably, when N was set to 1, coefficients regularisation was not applied; instead, simple regression was adopted, and thus the results show the performance of 30 randomly chosen univariate models (in-sample accuracy—M_RMSE_ = 0.74, SD_RMSE_ = 0.02; out-of-sample accuracy—M_RMSE_ = 0.98, SD_RMSE_ = 0.13). As the number of colour predictors increases, the EN mode exhibited a downward trend and smaller variations in both training and testing RMSE, suggesting an increase in model accuracy with the addition of more predictors. The in-sample RMSE of the OLS model also showed a consistent decrease. The results suggested the superiority of the combined model using multivariate approaches over any univariate models. On the other hand, the out-of-sample RMSE of the OLS model showed a consistent increase with more variables included and also large deviations across 30 random iterations. The larger divergence between in-sample and out-of-sample RMSE with the increase in predictor numbers indicated more serious overfitting problems of the OLS model as more variables were included. The large deviations also suggested the model performance was largely dependent on the selection of variables, whereas these issues had been effectively mitigated by the EN model.

## 4. Discussion

In this study, a novel four-step analytical framework was provided for modelling facial attractiveness from various colour characteristics (see Figure 1). It was the first time that a diverse set of colour predictors derived from realistic skin models were collectively considered. Correlation analysis was employed to refine variable selection and thus improve the efficiency of modelling. Multivariate approaches were then applied to assess all the relevant colour characteristics simultaneously and manage the complex data structure with a large number of correlated colour features. Due to the inherent correlations among colour variables (see Figure 3), conventional regression methods such as OLS may cause problems of multicollinearity and result in model overfitting (see Figure 6). In the current research, this issue was addressed by comparing three different multivariate approaches fit for high-dimensional datasets and using a novel testing dataset for model evaluation.

An ideal regression model for attractiveness prediction should be sparse, interpretable, and well predictive. The subset selection methods directly used the colour variables for prediction, which was straightforward and easy to interpret. However, the methods are largely affected by multicollinearity. In particular, the backward method, as it contained more variables than the forward method, resulted in an out-of-sample predictive error almost three times larger than the in-sample error (RMSE values). Moreover, the results could vary based on the order of the variable selection, which made it difficult to identify the most important contributor in the model (see Table 3). The PCR, PLSR, and RR have been identified as suitable algorithms to deal with multicollinearity [35,37]. Here, PCR and PLSR showed a relatively closer in-sample and out-of-sample accuracy and had the least number of predictors (PCR selected the first two principal components and PLSR only selected the first component) and thus the least degrees of freedom. The shortcoming was a certain amount of colour information was lost during the dimension reduction process. Such losses influenced the R^2^ values, resulting in both the PCR and PLSR models having the lowest variance in attractiveness (42.6% and 44.8%, respectively) compared to all the other methods. Dimension reduction techniques also have the disadvantage that they are difficult to interpret. Though the model only contains a small number of principal components, any future prediction will require the analysis of all the relevant colour variables to calculate those principal components. The regularisation regression models, LASSO and EN, were easier to interpret, as they performed feature selections. Both models gave relatively accurate predictions on the testing dataset, while EN performed better in terms of both predictive error (RMSE) and variance explained (R^2^). RR also gave a similar performance to EN but it was more complex, with all the colour predictors remaining in the model. Complex models are not necessarily performing better than simpler ones [61]. With fewer colour variables selected, the model was also easier to interpret and more practical to implement. In summary, considering the predictive accuracy, simplicity, and interpretability, the EN model with ML techniques was most recommended for modelling attractiveness from facial colour traits. For evaluating the overall performance of different algorithms in the future, all three criteria need to be taken into account, and determining the specific regression algorithm depends on the investigatory priority.

Relying on the appropriate techniques and algorithms, the predictive accuracy of attractiveness based on facial colour traits could be improved with a larger number of variables. Based on the best-fit EN regression, the multivariate models with more relevant colour predictors not only showed higher predictive accuracy (out-of-sample RMSE = 0.66) than the univariate models (out-of-sample RMSE = 0.98) but also effectively mitigated the issue of overfitting in classic models (see Figure 6). Recent research has criticised the univariate approach because manipulating a single variable while holding others constant prevents the assessment of the role of different colour cues as a whole in the attractiveness judgements of real faces [24,25]. Other studies based on realistic skin models showed only a limited role of colour in preference judgements [13,18,19,20,21]. Our findings, however, suggested that the multivariate approach based on realistic conditions could also underestimate the importance and reveal much weaker associations between skin colour and facial preference if only limited colour variables considered. The current approach, with a wide range of colour characteristics being studied, showed superior performance and confirmed the effectiveness of colour in attractiveness modelling. On the other hand, a few studies used multivariate approaches to build facial attractiveness models based on structural facial features including averageness, dimorphism, and symmetry, and their out-of-sample RMSE varied from 0.46 to 0.77 [62,63]. Compared to those studies, the colour-based models in this study showed a comparable importance of the colorimetric facial traits in attractiveness judgement among Chinese individuals.

Following the proposed framework, the most important colour traits for Chinese populations were also revealed. According to the complete correlation matrix (Figure 3), all the relevant colour predictors at significant levels were related to L*, a*, or h_ab_ without an exception. These results revealed that Chinese observers relied more on colour cues related to skin lightness (L*), redness (a*), or hue angle (h_ab_) for attractiveness judgement. The other two colour attributes, yellowness (b*) or chroma (C*), are less important and almost entirely unused to make decisions. Among these relevant colour predictors, the variable ranking of RR, LASSO, EN, and PLSR showed a large overlap. LASSO and EN selected the same top eleven colour predictors. Within these 11 colour predictors, the top 10 from RR, the top 9 from PLSR, the top 6 from PCR, the top 6 from SF, and the top 8 from SB were included (Table 3). After grouping the correlated variables (Figure 5), the most important features were identified: the brows colour contrast (a*), skin colour variation (overall or cheek), local skin hue angle (forehead, chin, or nose), the mouth colour contrast (a*), and local skin lightness L* (nose, chin, or cheek). The results were consistent with previous research on a part of the variables [7,13,17,22], but, more importantly, revealed the significance of assessing a wide range of facial colour characteristics to obtain a comprehensive estimate of the role of colour features on aesthetic preferences. Two new predictors, local skin hue angle and local skin lightness, were added to the analysis and found as important colour parameters for the first time. The results verified the assumption that h_ab_ is an important colour predictor for Chinese to evaluate attractiveness. Calculating the skin lightness of some local skin areas might be enough for future attractiveness predictions, instead of analysing the overall facial lightness.

The analytic framework proposed in this study offers a robust foundation for future research into the modelling of facial attractiveness. Any new facial colour characteristic that emerges as an influential factor in determining attractiveness can be added as a new colour predictor from the first step of variable identification. Alternate regression techniques might also be explored in the modelling phase to potentially enhance the model’s performance. Furthermore, this versatile framework can be expanded to investigate other facial impressions or preference attributes, and prediction models can be created accounting for variations in ethnicity, age, gender, and more. For practical implementation, our recommendation is to prioritise key colour features and combine them effectively, consider target populations and expert insights for specific applications, continuously update and validate the model, and integrate face recognition technology to achieve real-time feedback [64,65]. By concentrating on these crucial aspects, industries can utilise predictive models for facial attractiveness more efficiently, thereby enhancing their products and services.

## 5. Conclusions

The current study presented a complete and repeatable analytical framework for attractiveness modelling from various facial colour cues derived from realistic skin models. Within the framework, it was possible to evaluate the role of diverse facial colour cues holistically in attractiveness judgements of real faces. Based on the comparisons of various multivariate regression techniques, ML techniques with feature selection were recommended for future modelling. The proposed methodology with the best-fit model achieved high out-of-sample accuracy with a large number of colour predictors, while simultaneously effectively addressing the challenges related to multicollinearity and overfitting. Based on the methodology, the most important colour features influencing facial attractiveness judgments among Chinese individuals were identified. Our findings also demonstrated the importance of colour in facial attractiveness judgements, which could be comparable to those of facial structural features.

## Figures and Tables

**Table 2:** Comparison of the eight multivariate regression algorithms based on the RMSE and R^2^ for the training dataset and the testing dataset.

Algorithms	Training RMSE	Training R^2^ (%)	Testing RMSE	Testing R^2^ (%)
OLS	0.42	73.9	1.35	10.8
**Subset selection**				
SF	0.51	61.2	0.82	38.5
SB	0.44	71.4	1.17	12.0
**Dimension reduction**				
PCR	0.62	42.6	0.71	39.9
PLSR	0.61	44.8	0.68	39.6
**Regularisation**				
RR	0.60	51.8	0.67	43.5
LASSO	0.54	58.1	0.70	39.4
EN	0.55	56.9	0.66	41.8

**Table 3:** Ranking of the eleven colour predictors selected by LASSO and EN in the eight regression models. Variables that were not selected by SB or SF regression are marked as 0.

LASSO & EN Selected	OLS	SF	SB	PCR	PLSR	RR	LASSO	EN
Brows_C_a*	5	4	3	8	5	1	1	1
MCDM	12	6	4	9	11	3	2	2
Nose_h_ab_	7	0	6	18	7	2	6	3
MCDM_Cheek	10	7	0	15	17	4	7	4
Mouth_C_a*	17	0	7	10	2	5	8	5
Chin_L*	13	10	0	4	3	6	9	6
Forehead_h_ab_	4	5	0	19	9	11	5	7
Chin_h_ab_	8	0	0	21	14	10	3	8
Cheek_L*	2	2	2	3	1	7	11	9
Nose_L*	16	8	5	6	8	12	4	10
Mouth_C_h_ab_	15	9	0	16	10	9	10	11

## Data Availability

The data associated with this research will be available at https://www.kaidaxiao.co.uk/ (accessed on 5 January 2024).

## Supplementary Materials

The following supporting information can be downloaded at: https://www.mdpi.com/article/10.3390/s24020391/s1, Figure S1: Model performance of the (a) OLS, (b) SF, (c) SB, (d) PCR in predicting facial attractiveness for the training data (left column) and the testing data (right column); Figure S2: Model performance of the (e) PLSR, (f) RR, (g) LASSO, (h) EN in predicting facial attractiveness for the training data (left column) and the testing data (right column).
