# Supplementary material for: Predicting Facial Attractiveness from Colour Cues: A New Analytic Framework
Source: Sensors (Basel). 2024 Jan 9;24(2):391. doi: 10.3390/s24020391 (PMC10819822; doi:10.3390/s24020391)
Supplement: Supplementary file 1 [file sensors-24-00391-s001.zip › sensors-2756930-supplementary.pdf]

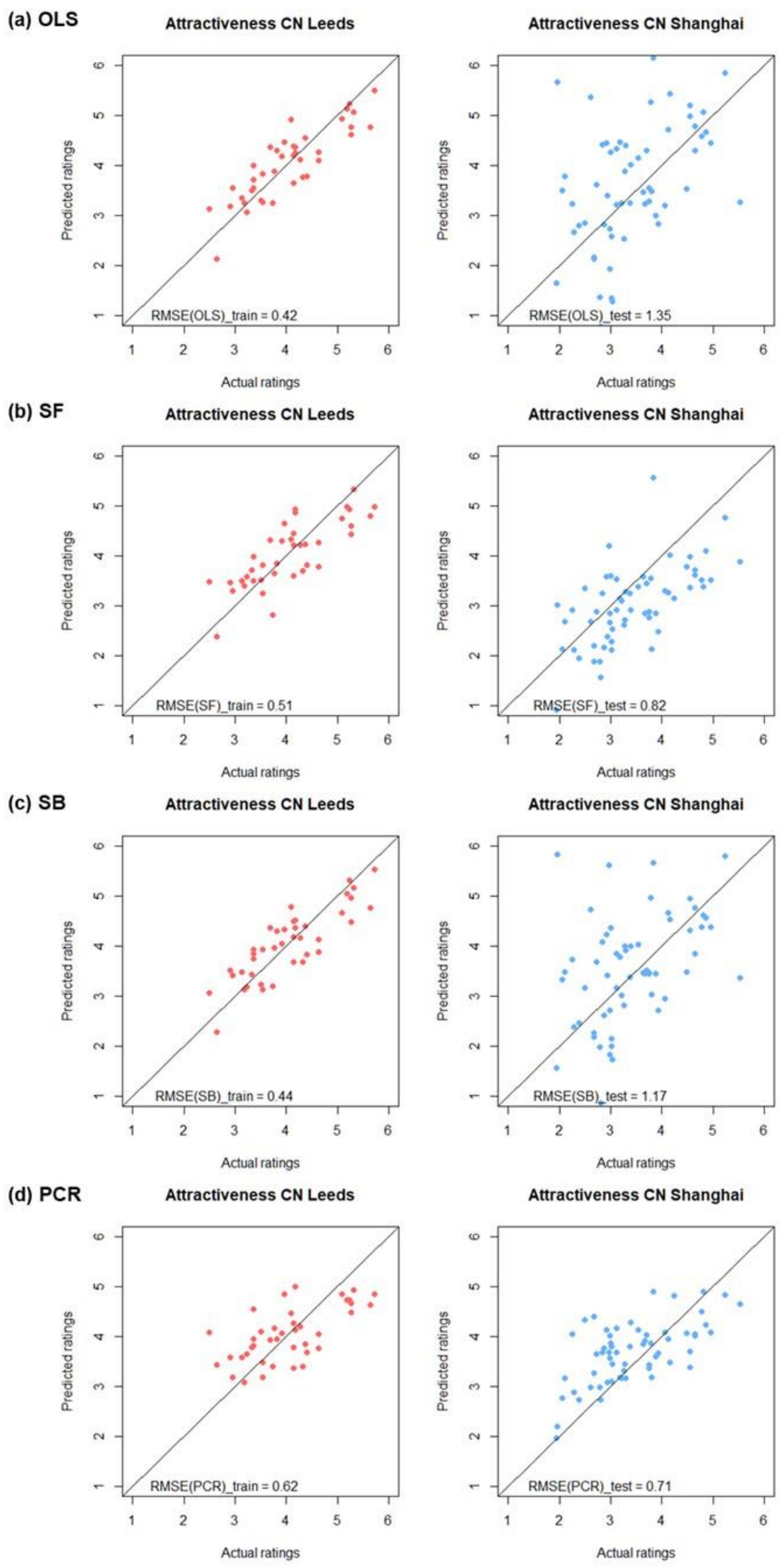

Figure S1: Model performance of the (a) OLS, (b) SF, (c) SB, (d) PCR in predicting facial attractiveness for the training data (left column) and the testing data (right column).

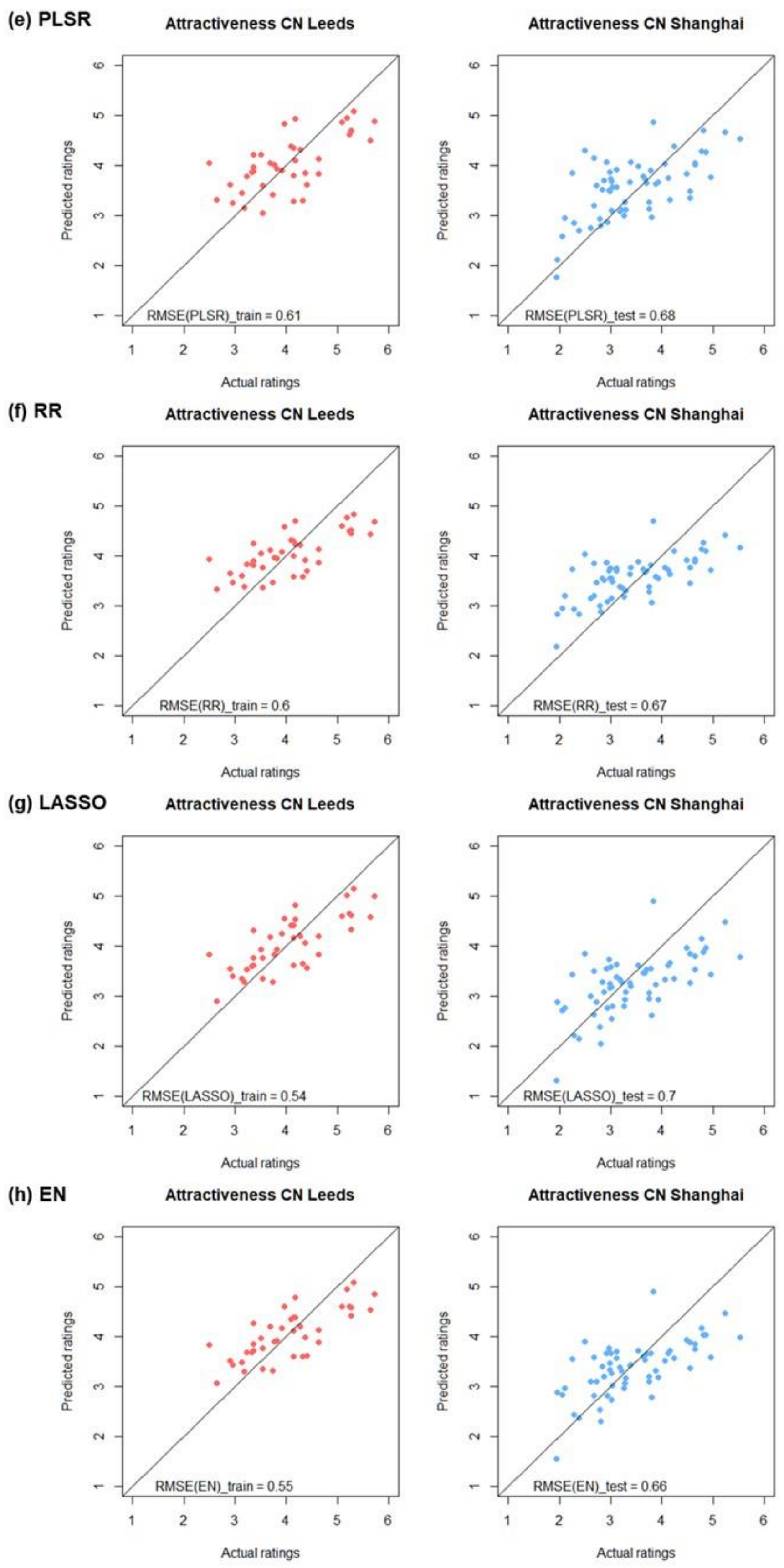

Figure S2: Model performance of the (e) PLSR, (f) RR, (g) LASSO, (h) EN in predicting facial attractiveness for the training data (left column) and the testing data (right column).
